# Supplementary figures and images for: Depressive symptoms exacerbate disability in older adults: A prospective cohort analysis of participants in the MemAID trial
Source: PLoS One. 2022 Nov 29;17(11):e0278319. doi: 10.1371/journal.pone.0278319 (PMC9707770; doi:10.1371/journal.pone.0278319)

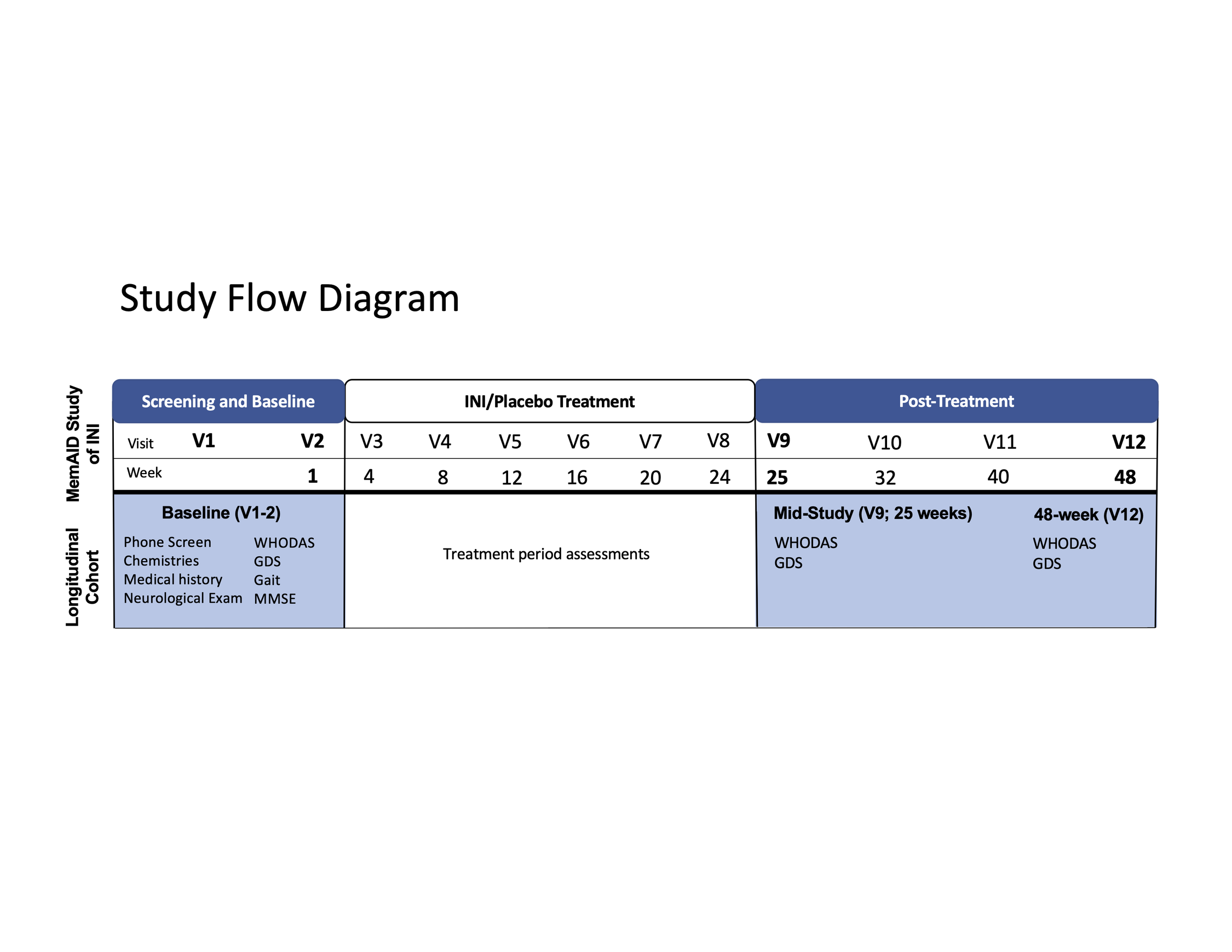

Supplement: S1 Fig — All participants included in the present study were concurrently enrolled in the MemAID clinical trial of intranasal insulin. Only data included in the longitudinal analysis are shown and were drawn from visits at baseline, 25 weeks, and 48 weeks. Additional assessments which were performed as part of the MemAID trial have been previously published (Novak, et al., Journal of Neurology 2022) and are not shown in the figure. INI: intranasal insulin; MMSE: Mini mental state examination; WHODAS: World Health Organization Disability Assessment Schedule 2.0; GDS: Geriatric Depression Scale. (TIF) [file pone.0278319.s001.tif]

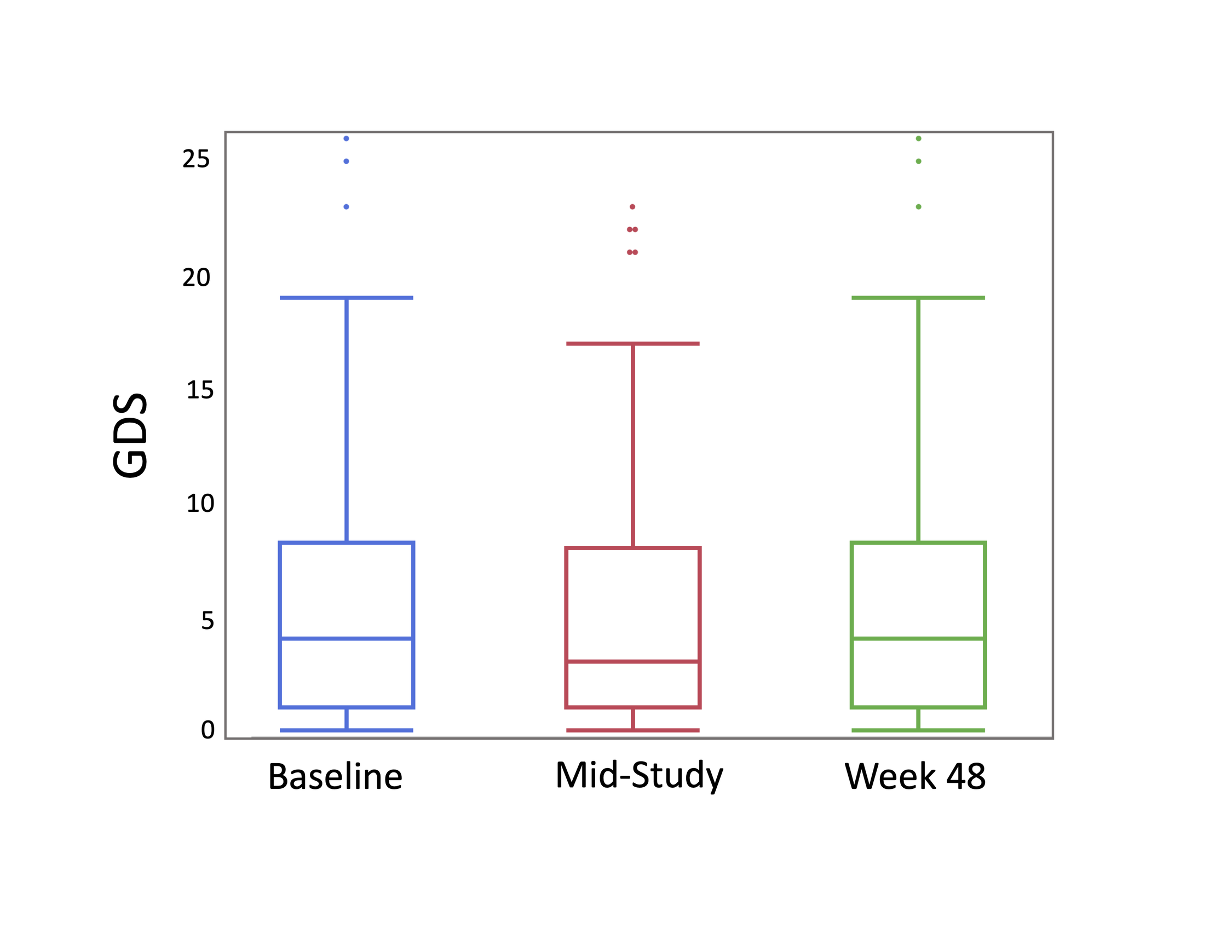

Supplement: S2 Fig — Mean depressive symptoms over the duration of the study are shown. At the group level, depressive symptoms were stable between baseline, Mid-study (week 25), and end of the study (week 48). GDS: Geriatric Depression Scale. (TIF) [file pone.0278319.s002.tif]
