# Supplementary material for: Depressive symptoms exacerbate disability in older adults: A prospective cohort analysis of participants in the MemAID trial
Source: PLoS One. 2022 Nov 29;17(11):e0278319. doi: 10.1371/journal.pone.0278319 (PMC9707770; doi:10.1371/journal.pone.0278319)
Supplement: S1 Appendix — Cross-sectional mediation analysis. Evaluation of baseline data showed that the mediation effects seen in the longitudinal analysis were already present at baseline. Post-hoc T2DM Subgroup analysis. The group of participants with T2DM showed more disability than controls, however, markers of diabetes severity were not related to greater disability. (DOCX) [file pone.0278319.s003.docx]

**S1 Appendix**

**Cross-sectional Mediation Analyses**

**Methods**

A cross-sectional mediation analysis was also performed to determine if the relationships seen in the longitudinal analysis were already seen at baseline, which had a larger sample size of 223 participants. A fixed-effects linear model tested the relationship between GDS and WHODAS at baseline, with covariates of age, sex, education, hemoglobin A1c (HbA1_C_), and race. Three separate parallel fixed-effects linear models tested for a mediation effect of GDS on the relationship between objective measures of functionality (MMSE, NW, and CCI) and WHODAS at baseline. Covariates for the MMSE and NW models were age, sex, education, HbA1_C_, and race. For the CCI model only education and race were included as covariates, since age, sex, and history of T2DM were already included in the CCI score. To test for mediation effects, GDS was added to each linear model in the manner described in Methods: Longitudinal Mediation Analysis and Direct and Indirect effects were calculated for each model.

**Results**

When GDS was added to the primary cross-sectional model for MMSE (Results: Cross-sectional Analyses), the R^2^_adj_ increased to 0.47. Higher HbA1_C_ (B=1.57, *p*=0.002) remained a predictor of WHODAS, but MMSE was no longer a significant predictor (B=-0.52, *p*=0.155). GDS showed a partial mediation effect, with an Indirect effect of 49.9% of the Total (Table S1).

**S1 Table: Mediation effect of depression on the relationship between gait speed, cognition, and medical comorbidities on disability at baseline**

| **Predictor of Functionality** | **Primary Model** | | | **Model with GDS** | | | **Change in B** | **Direct Effect** | **Indirect Effect** |
| --- | --- | --- | --- | --- | --- | --- | --- | --- | --- |
|  | B | d.f. | p-value | B | d.f. | p-value | % change | % of Total | % of Total |
| Cognition (MMSE) | -1.01 | 222 | 0.027 | -0.52 | 222 | 0.155 | 48.5% | 50.1% | 49.9% |
| Gait Speed (NW) | -0.14 | 221 | <0.001 | -0.09 | 221 | 0.002 | 35.7% | 63.1% | 36.9% |
| Comorbidities (CCI) | 1.77 | 222 | <0.001 | 1.29 | 222 | <0.001 | 27.1% | 69.9% | 30.1% |

**S1 Table Legend: Results of cross-sectional mediation analysis.** The effect size of each predictor (NW, MMSE, and CCI) on disability is shown for each model used in the cross-sectional mediation analysis. A change in B > 10% after GDS is added to the model supports a mediation effect of GDS. The Direct effect represents the proportion of the effect of each predictor on disability, independent of GDS. The Indirect effect represents the proportion of the total effect attributable to mediation by GDS.

When GDS was added to the primary cross-sectional model for NW, the R^2^_adj_ increased to 0.49, and slower NW (B=-0.09, *p*=0.002), higher GDS (B=1.30, *p*<0.001), and higher HbA1_C_ (B=1.33, *p*=0.007) were all predictors of WHODAS. GDS showed a partial mediation effect, with an Indirect effect of 36.9% of the Total (Table S1).

When GDS was added to the primary cross-sectional model for CCI, the adjusted R^2^ of the model increased to 0.48. CCI (B=1.29, *p*<0.001), GDS (B=1.35, *p*<0.001), education (B=-0.51, *p*=0.006), and race (B=2.37, *p*=0.002) were all predictors of WHODAS. GDS showed a partial mediation effect, with an Indirect effect of 30.1% of the Total (Table S1).

**Post-hoc T2DM Subgroup Analysis**

**Methods**

We compared all variables of interest (GDS, medication for depression, WHODAS) between T2DM and control participants using 2-tailed t-tests for continuous variables and a Fisher’s exact test for categorical variables.

Then, we investigated if T2DM severity or treatment were related to longitudinal disability outcomes. In T2DM participants, a mixed-effects linear model tested the effects of hemoglobin A1c (HbA1_C_), oral medications for diabetes, and insulin use (independent variables) on WHODAS longitudinally (dependent variable). The model included a main fixed effect of time, defined as average days from baseline of each visit, crossed-random effects for subject and subject and time interaction to account for repeated measures, and covariates of GDS, use of medications for depression, age, sex, treatment group, education, and race.

**Results**

At baseline, T2DM participants had worse disability than controls (*p*<0.001, mean WHODAS: T2DM 16.6, controls 7.8) and more depressive symptoms (*p*<0.001, mean GDS: T2DM 6.9, controls 4.5). Antidepressant usage was not different between groups.

In the T2DM mixed effects model, greater disability was associated with GDS (B=1.27, *p*<0.001) and race (B=1.69, *p*=0.046). There was no significant effect of HbA1_C_ or use of oral medications for diabetes or insulin.
