# Supplementary material for: Depressive symptoms exacerbate disability in older adults: A prospective cohort analysis of participants in the MemAID trial
Source: PLoS One. 2022 Nov 29;17(11):e0278319. doi: 10.1371/journal.pone.0278319 (PMC9707770; doi:10.1371/journal.pone.0278319)
Supplement: Attachment — Submitted filename: Comments and recommendation.docx [file pone.0278319.s004.docx]

Dear authors! Thank you for conducting this study, which might also assist mental health professionals in recognizing and treating depression symptoms in the elderly population to reduce disabilities related to depression symptoms . However, your methods and materials weren't quite clear. I've stated my review comments below, and each one should be addressed as appropriate.

1. Abstract: Has all scientific information and it is clearly written.

But

- Line 29-31 –You have been prospectively analyzed data from 223 adults (age 50-85) over 48 weeks who were participating in a clinical trial “Memory Advancement by Intranasal Insulin in Type 2 Diabetes. But in this manuscript, line 89 - only 106 study participants were type2 Diabetes mellitus patients (T2DM). these two phrase contradict to each other , it needs correction
- In the abstract part, you stated that study participants' cognition, walking speed, and medical disabilities were assessed at baseline, but it is not clear how and by what medical diagnostic workup, cognition, walking, and medical comorbidities were assessed. Furthermore, what about study participants' status regarding their baseline cognition, walking speed, and medical comorbidities? I think all these need a brief and clear description.
- Line 31& 32 - The World Health Organization Disability Assessment Schedule 2.0 (WHODAS) measured disability. And line 33 & 34 -WHODAS were assessed at Baseline and at 8-week intervals	- it seems like redundancy and need	revision in one line
- Better to revise and rewrite Key words, considering MeSH (Medical subject headings) terms rather than repeating words in introduction.
- You have been investigated mood as an important modifiable risk factors that my mediate the effect of physical and cognitive decline on disability. But, it is obvious that mood is a broad term which can be characterized by individual’s subjective feelings that might be normal, depressed, irritable, and expansive and etc. And sometimes it can be a gate symptom for manic episode. So it is beyond your study title. The researchers should justify this fact, why they were used broad term “mood” as important modifiable risk factors.

2. Introduction

- Line 54 -what is the recent global total estimated number of your study population (50–85-years-old)? Furthermore, it is preferable to describe the total number of elderly people in your study area/setting.
- It would be better if the introduction part of this study had more focus and described the magnitude of depression symptoms' impact on disabilities among the elderly population.
- Line -55 -is not complete sentences and does not give sense

3. Methods and materials

- The section on ‘methods and materials’ in this manuscript weren’t written clearly or appropriately. As a result, it generally needs to be revised and rewritten in order to be understandable and scientifically sound. For instance, in “Study setting and design” part, line 79- Data for the present analysis were collected before and after the treatment period of intranasal insulin. Which is not appropriate and right way to write about data collection procedure in “ study setting and design” section
- I note that the Ethics approval information you provided in the ‘Participants section’, line 83-84 is not appropriate. Better if the researchers provide separate headings for ‘Ethics approval’
- Line 141-142, the primary outcome was WHODAS and exposures of interest were GDS, NW, MMSE, and CCI. WHODAS, GDS, MMSE, and CCI have been used to collect data related to dependent and independent variables/outcomes. So, WHODAS, GDS, MMSE, and CCI cannot be considered as primary out-comes. The author of this is study expected to justify this issue.
- Line 102-103 -The primary outcomes were measures of disability. What kind of disability? Is it physical, cognitive or other types of disability? it is not clear
- “Sample size” written next to “Post-hoc T2DM Subgroup Analysis”. This also not appropriate
- Sample Size Determination and Sampling Technique followed are not clear, better if clearly described.
- When this study was employed? Better if study period will be incorporated to the methodology section
- All the following subheadings, better to rewrite in	sentence case

Line 73 Materials and Methods can be correct as Methods and materials

Line 74 Design and Setting can be correct as Study setting and design

Line 106 Assessment of Disability and Functionality can be corrected as Assessment of disability and functionality

Line 116 Assessments of Mood and Cognitive Function can be corrected as Assessments of mood and cognitive function

Line 124 Assessment of Mobility can be corrected as Assessment of mobility

Line130 Assessment of Medical Comorbidities and 10-year Mortality Risk can be corrected as Assessment of medical comorbidities and 10-year mortality risk

Line 140 Statistical Analyses can be corrected as Statistical analyses

Line 146 Cross-sectional Analyses can be corrected as Cross-sectional analyses

Line 157 Longitudinal Analysis 157 of Change in Disability Can be corrected as Longitudinal analysis 157 of change in disability

Line 173 Longitudinal Mediation Analyses

Post-hoc T2DM Subgroup Analysis

Line 203 Sample Size

Line 209 Demographic and Clinical Variables

- Your study design is not clear and not in line with your study objective / title –it is stated as Participants were enrolled in the Memory Advancement with Intranasal 78 Insulin (MemAID) study, a randomized, double-blinded, placebo-controlled clinical trial 79 (ClinicalTrials.gov NCT02415556, FDA IND 107690).
- At the beginning why study participants were enrolled in the Memory Advancement with Intranasal 78 Insulin problems, did they have known diagnosed Memory problems? If so, better to describe briefly.
- The study design for your study title is not clear enough. Is it Crossectional, case-control RTC or prospective cohort study? It is not clear, because the following statements are stated as in - Line 79-80 –‘Data for the present analysis were collected before and after the treatment period of intranasal insulin, Line 88-89 -For the cross-sectional analysis, we used data from 223 participants enrolled in the MemAID trial (117 controls and 106 with T2DM) and Line-96-97-One participant who completed the MemAID trial did not 97 complete weeks 48 WHODAS so was excluded from the present analysis. Why one participant only excluded from your study and what about those 67 participants who have not been included in longitudinal analysis in you study? Better to justify
- At the beginning you stated that study participants were evaluated at base line, then at the intervals of every 8-weeks (i.e., 8, 16, 24, 32, 40 and 48 weeks of their participation in MemAID ) , but you did not stated/showed their evaluation of depression symptoms , cognitive , walking status and their disability status at each of these evaluation periods . It needs clear and brief description of participants evaluation results at 8, 16, 24, 32 , 40 and 48 weeks
- Some of your study results stated within Methods and materials section. For instance, line 153 to 154, since 78% of participants self-reported race as “White,” race was encoded as a dichotomous variable (White vs. Non-White).

4. Result

- The first column of Table 1, line 225, is unclear; are they variables? If so, categorization is recommended.
- What are the differences between the study participants' race and ethnicity classification as shown in Table 1?
- For clarification, some of the variables in column one of Table 1—including sex, ethnicity, years of education, and others—need further categorization.
- Table 1 shows the age^a^, (%) female) ^b^, (% Latinx) ^b^, and other variables. What are ^a^ and ^b^ represented in this table's column 1? Better to write Key notes under the table
- According to your study 175 participants did not show depressive symptoms at baseline. During your longitudinal depression evaluation by using GDS, how many of them developed depressive symptoms over 48 week’s period?

5. Discussion

- The authors discussed their study findings in depth which is scientifically appropriate and smart.
- It is better if the researchers provide separate headings and rewrite for “abbreviations and availability of data and materials “next to conclusions section
